# Repurposing Drugs in Oncology (ReDO)—itraconazole as an anti-cancer agent

**DOI:** 10.3332/ecancer.2015.521

**Published:** 2015-04-15

**Authors:** Pan Pantziarka, Vidula Sukhatme, Gauthier Bouche, Lydie Meheus, Vikas P Sukhatme

**Affiliations:** 1 Anticancer Fund, 1853 Strombeek-Bever, Belgium; 2 The George Pantziarka TP53 Trust, London, KT1 2JP, UK; 3 GlobalCures, Inc; Newton MA 02459, USA; 4 Beth Israel Deaconess Medical Centre and Harvard Medical School, Boston, MA 02215, USA

**Keywords:** drug repurposing, itraconazole, hedgehog pathway inhibition, ReDO project

## Abstract

Itraconazole, a common triazole anti-fungal drug in widespread clinical use, has evidence of clinical activity that is of interest in oncology. There is evidence that at the clinically relevant doses, itraconazole has potent anti-angiogenic activity, and that it can inhibit the Hedgehog signalling pathway and may also induce autophagic growth arrest. The evidence for these anticancer effects, *in vitro*, *in vivo*, and clinical are summarised, and the putative mechanisms of their action outlined. Clinical trials have shown that patients with prostate, lung, and basal cell carcinoma have benefited from treatment with itraconazole, and there are additional reports of activity in leukaemia, ovarian, breast, and pancreatic cancers. Given the evidence presented, a case is made that itraconazole warrants further clinical investigation as an anti- cancer agent. Additionally, based on the properties summarised previously, it is proposed that itraconazole may synergise with a range of other drugs to enhance the anti-cancer effect, and some of these possible combinations are presented in the supplementary materials accompanying this paper.

## Current usage

### Introduction

Itraconazole (ITZ) is a triazole anti-fungal treatment widely used in the prevention and systemic treatment of a broad range of fungal infections, including *aspergillosis*, *blastomycosis*, *candidiasis*, *histoplasmosis*, and in some dermatological and nail infections. The mechanism of action for this antifungal activity is through the decrease of ergosterol synthesis, required for membrane integrity of fungal cells, via inhibition of the lanosterol 14 alpha-demethylase (14DM) catalyst. Immunocompromised patients are often treated prophylactically with triazole anti-fungal drugs, including ITZ, particularly if there is a risk of *aspergillosis*. ITZ is commonly available as a generic, prescription-only drug. Common trade names include Sporanox (Janssen) and Onmel (Merz).

### Dosage

ITZ is most commonly administered orally, either as 100 mg or 200 mg capsules or as oral solution. It can also be administered intravenously, though this route is less commonly used. Dosing varies by indication; generally it is used in the range 100 mg–600 mg daily, for between one to 30 days. It is also used for long-term maintenance or prophylaxis, for example 200 mg–400 mg daily for HIV-infected patients, 400 mg daily for patients suffering from chronic pulmonary aspergillosis, or 100 mg per day for more than one year for the treatment of paracoccidioidomycosis [1].

### Toxicity

ITZ is generally well-tolerated, though caution is advised with patients at high risk of heart failure or impaired hepatic function. The most common side effects of ITZ are nausea, abdominal pain, and rash. Less commonly, gastrointestinal upsets have been reported, (including vomiting, flatulence, diarrhoea, and constipation), headache, dizziness, and peripheral neuropathy. Rare but serious side effects have included liver failure, chronic heart failure, and neutropenia. ITZ is contra-indicated during pregnancy unless the risk of fungal infection is life-threatening; it is also not advised during breast feeding [2]. Doses are reduced for children, being in the range of 5–10 mg/kg/day, although for severe infections the maximum doses in children are equivalent to the adult doses (for example 400 mg/day for one year for the treatment of severe blastomycosis in children) [3].

### Pharmacokinetics

Bioavailability of ITZ is maximised by taking with food for the encapsulated form, or on an empty stomach for the oral solution. A single 200 mg dose, as a capsule with food, produces an average peak plasma concentration of 239 ng/mL (0.34μM) within 4.5 hours, whereas at steady state (after 14 days of 200 mg every 12 hours), the average plasma concentration is 1881 ng/mL (2.67 μM) [4]. While peak plasma concentrations and elimination half-life are similar with the capsule form, the oral solution has approximately 30% greater overall bioavailability as measured by area under the curve (AUC) [5]. The plasma half-life of 200 mg of the capsule form is 24 hours at steady state. The mean absolute bioavailability is around 55%, and as a highly lipophilic molecule ITZ has a high affinity for tissues, achieving concentrations two to ten times higher than those in plasma [6, 7].Oral absorption of ITZ is reduced when gastric acid production is decreased, thereby caution is advised for patients taking H_2_ receptor agonists (H_2_RAs) (e.g. cimetidine or ranitidine) or proton pump inhibitors (e.g. omeprazole or pantoprazole).

ITZ undergoes extensive first-pass hepatic metabolism, with unchanged drug barely detectable in urine and between 3–18% of the dose given detectable as the parent drug in the faeces of healthy volunteers. Hydroxyitraconazole also has anti-fungal activity [8]. ITZ is a potent inhibitor of cytochrome P450 (CYP) 3A4, and there is an extensive list with regard to drug interactions [9]. In the context of cancer treatment, it should be noted that there are a number of credible case reports that concomitant ITZ increases the severity of neurotoxicity associated with vincristine [10, 11].

Overall there is a high-degree of inter and intra-patient variability in plasma concentrations, with differences influenced by drug formulation (oral solution or tablets), changes in kinetics during long-term treatments, interactions with food intake, and other medications and changes in pathological status of patients. It is generally recommended, therefore, that for long-term treatment patients be regularly monitored for plasma levels [6, 12, 13].

## Pre-clinical evidence in cancer—*in vitro* and *in vivo*

Early pre-clinical investigation of the anticancer potential of ITZ focused on a potential role as a potentiator for chemotherapeutic drugs, particularly as a possible agent to reverse multi-drug resistance (MDR).Examples include *in vitro* studies on the reversal of MDR in a murine P388 leukaemia cell line resistant to daunorubicin [14], human leukaemia cell lines resistant to Adriamycin and etoposide [15] and in human breast cancer resistance protein (BCRP) expressing human embryonic kidney (HEK) cells resistant to topotecan [16]. Of note these results were achieved using clinically achievable doses of ITZ, a finding that was later confirmed in clinical studies (see next section).

In addition to reversing MDR action, other pre-clinical studies have indicated that ITZ has a range of activities of value in an anti-cancer context. A drug screen of a large panel of FDA-approved drugs in a human umbilical vein endothelial cell (HUVEC) proliferation assay indicated that ITZ had potent anti-angiogenic activity [17]. The *in vitro* screen was confirmed *in vivo* using a murine Matrigel model, which showed that the mice treated with an IV dose of ITZ equivalent to a typical human dose showed a 67.5% decrease in new vessel formation compared to control mice. Analysis showed that the levels required for an anti-angiogenic response could also be achieved using a 200 mg oral dose of the drug.

Subsequently, the same research group performed a screen for repurposed drug combinations with anti-angiogenic activity. This screen of 741 drug combinations identified ITZ and the immunosuppressant cyclosporin A as a potent and synergistic anti-angiogenic combination [18].

The anti-angiogenic activity of ITZ was later investigated in a panel of non-small cell lung cancer (NSCLC) cell lines (NCI-H358, NCIH1838, NCI-H596, and NCI-H1975) and two primary NSCLC xenograft models [19]. ITZ was shown to inhibit proliferation of HUVEC, but there was no evidence of a direct anti-proliferative effect on NSCLC cells. ITZ, alone and in combination with cisplatin, inhibited growth of primary NSCLC xenografts, increased expression of HIF1α, and reduced tumour vascular area compared to vehicle-treated controls.

A drug screen also identified ITZ as an inhibitor of the Hedgehog pathway at a clinically relevant concentration of 800 nM [20]. Interestingly, the primary metabolite (hydroxyitraconazole), was also shown to be an inhibitor of Hedgehog signalling. In addition to *in vitro* analysis, an *in vivo* model of murine Hedgehog-dependent medulloblastoma showed that ITZ treatment at 100 mg/kg b.i.d. or 75 mg/kg b.i.d., both reduced tumour growth. The same study also used a murine basal cell carcinoma (BCC) model, and treatment with ITZ suppressed tumour growth. When treatment was interrupted BCC tumours recommenced growth. The authors estimated that the human doses of ITZ required to achieve the serum levels achieved in these murine models is in the range of 600–900 mg/day, a high dose but one which has been used clinically for long periods in humans [21].

Further work by the same group has shown that ITZ, along with arsenic trioxide (ATO), is able to reverse resistance to existing Hedgehog inhibitors being tested in clinical trials (the cyclopamine analogue IPI-926 and cyclopamine competitive binding agents such as vismodegib, NVP-LDE225, and XL-139) [22]. Using BCC and medulloblastoma mouse allograft models resistant to mutant Hedgehog signalling, the authors showed that ITZ and ATO either singly and in combination were effective against resistant tumours.

Hedgehog signalling was also investigated in a panel of eight malignant pleural mesothelioma (MPM) cell lines *in vitro* [23]. After showing that Hedgehog signalling was upregulated in the cell lines, the authors tested four pathway inhibitors: vismodegib, ITZ, ATO, and the investigational agent GANT61. Results showed that ITZ was effective in all eight cell lines with IC_50_< 5 μM.

Investigation into the anti-cancer effect in glioblastoma has found that ITZ treatment, both *in vitro* and *in vivo*, causes dose dependent growth arrest but not apoptosis in U87 and C6 glioblastoma cells and a xenograft mouse model [24]. Analysis shows that this growth inhibition is related to induction of autophagy, and that the inhibition of autophagy reverses the effect of ITZ. Induction of autophagy is shown to be related to inhibition of the AKT-mTOR pathway, possibly related to ITZ-induced changes in cholesterol trafficking.

Evidence has also been produced that indicates that clinicians may need to exercise caution in the use of ITZ therapy in patients being treated with monoclonal antibodies. The monoclonal antibody rituximab is an effective therapy for diseases characterised by CD20-expressing B cell dysfunction or over-expression, including lymphomas, leukaemias, and auto-immune conditions. Concurrent use of ITZ was shown, *in vitro* and *in vivo* using a murine xenograft model of lymphoma, to abrogate the therapeutic effect of rituximab [25]. *In vitro* analysis suggested that this effect of ITZ was specific to lipid-raft-associated molecules, and ITZ impaired alemtuzumab-induced cell death in a dose dependent manner.

## Human data

In addition to the extensive pre-clinical data outlined above, there has also been a range of clinical studies performed to assess the therapeutic effects of ITZ in cancer. Note that these are studies looking specifically at the anti-cancer effect of ITZ; studies and trials which have assessed the impact of ITZ, as a CYP 3A4 inhibitor, on the pharmacokinetics of *other* anticancer agents have not been included. Also not included are a number of studies of ITZ as an anti-fungal prophylactic in cancer patients undergoing treatment.

Based on the findings that ITZ could potentially reverse resistance to daunorubicin in a murine leukaemia cell line [14], Vreugdenhil and colleagues analysed data from a double-blinded randomised clinical trial of ITZ as an anti-fungal prophylactic in neutropenic leukaemia patients treated with daunorubicin [26]. The analysis included 23 patients with acute lymphoblastic leukaemia (ALL), of whom 11 received ITZ, and 42 patients with acute myeloid leukaemia (AML), of whom 17 received ITZ. Results showed that in ALL patients, disease-free survival (DFS) tended to be longer in the ITZ group as compared to control (P < 0.06), but the remission rate was not significantly different. In the AML patients, DFS and remission rates were similar in the ITZ and control groups.

A phase II non-comparative randomised study investigated two dose schedules of ITZ monotherapy in men with castration-resistant metastatic prostate cancer [27]. The low dose (200 mg/day) arm closed early after accruing 17 patients because of a pre-specified futility rule. The high dose arm (600 mg/day) completed, and accrued 29 patients. The primary endpoint was the prostate-specific antigen (PSA) progression-free survival (PFS) rate at 24 weeks, with a 45% success rate required to achieve statistical significance. Results showed that at 24 weeks the PFS rate was 11.8% for the low dose arm, and 48% for the high dose arm. Median PFS, a secondary endpoint, was 11.9 weeks and 35.9 weeks in the two arms respectively. The PFS value of 35.9 weeks is of a range similar to other investigational drugs in this patient population. One patient in the low dose arm and two patients in the high dose arm experienced partial response according to response evaluation criteria in solid tumors (RECIST) criteria. Of note, ITZ treatment also had positive effects on circulating tumour cell counts and Hedgehog signalling was shown to be reduced in skin biopsy samples. No changes were reported for plasma vascular endothelial growth factor (VEGF) levels in either arm. Toxicity was greater in the high dose arm, with the most common side effects being fatigue, nausea, anorexia, rash, and a syndrome of hypokalaemia, hypertension, and oedema. Although there were no grade 4 toxicities, 4 (14%) patients in the high dose arm came off study because of toxicity (one not drug related). There was no negative impact on testosterone levels in either treatment arm.

A case report has also been published by the same group [28]. A 65-year old patient with biochemically recurrent prostate cancer unwilling to undergo castrating treatment was treated with high dose ITZ (300 mg b.i.d.). Treatment with ITZ caused a >50% reduction of PSA level after 12 weeks with no significant change in testosterone level. However, treatment was stopped after five months because of elevated bilirubin levels (which returned to normal range on treatment cessation). The 50% decrease in PSA levels was maintained during ITZ treatment but increased after ITZ discontinuation.

The pre-clinical evidence of anti-angiogenic activity in NSCLC was followed by a phase II trial as a second line therapy in metastatic nonsquamous NSCLC [29]. Patients were randomised to pemetrexed(PM) with or without ITZ (PM+ITZ) at a dose of 200 mg/day, on a 21-day cycle, with PM 500 mg/m_2_ on day 1, and ITZ daily. Treatment was intended to last until disease progression, although the trial had to complete early because of the increased usage of PM in a first-line setting. The intended accrual had been 112 patients, and primary end points included the progression-free survival rate at three months. The number of patients actually enrolled was 23, with 15 randomised to PM+ITZ, and 8 to PM alone. At three months, the PFS rate for PM+ITZ was 67%, versus 29% on the PM arm (P = 0.11). Median PFS was 5.5 months for PM+ITZ compared to 2.8 months for PM (hazard ratio = 0.399, P = 0.089). Median overall survival (OS) for patients receiving PM+ITZ was 32 months versus eight months for PM (hazard ratio = 0.194, P = 0.012). Toxicities were equivalent in the two arms. While the small sample size makes it difficult to draw firm conclusions from the results, the authors report an intention to launch a future phase II trial in a more relevant clinical context–cisplatin and gemcitabine with and without ITZ, with additional end-points including assessments of tumour blood flow and hypoxia.

Results have also been reported for a small phase II trial in basal cell carcinoma (BCC) [30]. In this small open label trial, two cohorts of patients were treated either with 200 mg b.i.d. for one month (15 patients), or 100 mg b.i.d. for an average of 2.3 months (four patients). Primary end points were changes in proliferative and Hedgehog-related biomarkers. Secondary end points included change in tumour size for a subset of patients with multiple non-biopsied tumours. Results showed a reduction in tumour cell proliferation (as measured by Ki67 staining) of 45% (P = 0.04), Hedgehog pathway activity (as measured by GLI1 mRNA) of 65% (P = 0.03), and reduced tumour area of 24% (95% CI, 18.2–30.0%). In a subset of patients previously treated with the Hedgehog-inhibitor vismodegib, and in control patients, there were no changes in cell proliferation or tumour size. Of eight patients with multiple non-biopsied tumours, four achieved partial response, and four had stable disease. Toxicity was low: one patient withdrew because of grade 2 fatigue, and one patient withdrew because of congestive heart failure from previous chemotherapy with Adriamycin.

A retrospective analysis of patients with recurrent ovarian clear cell carcinoma treated with a combination of chemotherapy and adjunctive ITZ was performed by Inoue and colleagues [31]. Patients who had progressed during the first-line treatment or during platinum-based chemotherapy at recurrence and who continued chemotherapy were included. ITZ was used with the aim of potentiating the action of the chemotherapy drugs and was selected because of the preclinical evidence for ITZ inhibition of drug efflux and angiogenesis. Eight patients were identified who had received docetaxel and carboplatin with ITZ, (oral solution at a dose of 400 mg/day, days -2 to 2 on a two-week cycle). The response rate was 44%, median PFS was 544 days (95% CI = 82–544 days) and median OS was 1047 days (95% CI = 462–1332 days). The authors deemed the figures encouraging in a patient population with disease that rarely responds to chemotherapy. A subsequent analysis, also retrospective, looked at refractory ovarian cancer patients treated with adjunctive ITZ as part of second or later chemotherapy regimen [32]. Of 55 patients with refractory ovarian cancer, 18 received ITZ as part of a second or third line protocol. The ITZ dose was 400–600 mg, oral solution administered on days -2 to 2 or 3, on a two-week cycle. Patients not treated with ITZ received a range of second or later chemotherapies, including pegylated liposomal doxorubicin, gemcitabine, docetaxel, and other standard drugs. Overall, the chemotherapy response rate was 18% (10 of 55 patients), however, the response rate for ITZ was 32% (six patients of 19), whereas it was only 11% (four of 36) for those not receiving ITZ, (P = 0.06). In terms of PFS, median PFS for ITZ was 103 days (95% CI > 84 days) and 53 days for non-ITZ (95% CI = 38–88 days), (P = 0.014). Median OS for ITZ was 642 days (95% CI = 238–1166 days) and for non-ITZ it was 139 days (95% CI = 89–183 days), (P = 0.006). The ITZ hazard ratio was 0.24 (95% CI = 0.10–0.60; P = 0.002) for PFS and 0.27 (95% CI = 0.11–0.68; P = 0.006) for OS.

The same team also performed a retrospective analysis of heavily pre-treated triple negative breast cancer patients with recurrent disease [33].Thirteen patients were identified who had been treated with two or more lines of chemotherapy, 12 of whom had metastatic disease in the lungs, liver, or brain. All patients were subsequently treated with a regimen of docetaxel, carboplatin, and gemcitabine with adjunctive ITZ on a two week cycle. The ITZ dose used was 400 mg, administered on days −2 to 2 of the chemotherapy treatment. The response rate was 65% (95% CI = 35–88%). The median PFS was 10.8 months (95% CI, 7.6–15.3 months), and the median OS was 20.4 months (95% CI: 13.1–41.4 months), with data on three patients censored. Again, despite the low number of patients and the retrospective nature of the analysis, the authors deemed these results encouraging.

A pilot trial of ITZ pharmacokinetics in patients with metastatic breast cancer has also reported results. Patients (n = 14) received oral ITZ at a dose of 200 mg/day until disease progression or unacceptable toxicity. Primary outcomes were pharmacokinetic data, (plasma levels of ITZ and hydroxyitraconazole), correlated with measures of angiogenesis—plasma VEGF-A and thrombospondin -1 (TSP-1), serum basic fibroblast growth factor (bFGF), and placental growth factor PlGF—at baseline, two and four weeks. Median ITZ/hydroxyitraconazole levels at two and four weeks were 181/331.5 ng/mL and 202/337 ng/mL respectively. Median bFGF and PlGF levels decreased with administration of ITZ from baseline to weeks two and four. The bFGF displayed high correlations with ITZ and hydroxyitraconazole at weeks two and four although not statistically significant. Plasma TSP-1 increased at weeks two and four. VEGF-A levels increased from baseline to week two, but decreased with drug administration during weeks two to four. PlGF, TSP-1, VEGF-A did not correlate with drug levels. Of 13 evaluable patients, one had a partial resonse (PR), three stable disease (SD), and nine progressive disease (PD). Estimated time to progression and OS were 1.8 and 19.3 months respectively [34].

A report has also been published detailing a case of pancreatic cancer which showed response to ITZ treatment [35]. The patient was a heavily pre-treated 64-year- old male with unresectable stage III pancreatic adenocarcinoma who developed disseminated histoplasmosis following his third cycle of gemcitabine. He was treated with ITZ for nine months, without concurrent chemotherapy or other treatment, at which point his pancreatic cancer was reassessed and found to be resectable. Following successful surgical resection, the patient was followed for a period of four years and showed no signs of recurrence or metastatic disease. However, he later reported weight-loss and ill-health and a scan revealed a new primary cancer, shown to be NSCLC. The treating physicians assessed that the reduction in pancreatic tumour had been caused by the ITZ treatment.

Additionally, a case report exists of a patient suffering from mycosis fungoides, a common form of cutaneous T-cell lymphoma [36]. The patient, a 55-year old man, was treated with ITZ at 200 mg/day for seven days in case his symptoms were due to seborrhoeic dermatitis. Symptomatic improvement was noted within 3 days and all lesions had disappeared by the end of treatment. A subsequent recurrence was similarly treated with ITZ and again showed a response. The diagnosis of mycosis fungoides was made on the basis of histological features from repeated biopsies and lack of evidence of any fungal infection and the response to ITZ was therefore unexpected.

## Clinical trials

Data for clinical trials as assessed on 23rd February 2015.

NCT01787331—A phase II study of ITZ in biochemical relapse in prostate cancer. This single-arm trial is currently recruiting. Patients with non-castrate, non-metastatic, biochemically relapsed prostate cancer who have received prior definitive local therapy are prescribed oral ITZ at a dose of 600 mg/day (300 mg b.i.d.). The primary outcome measure is the proportion of men who experience a > 50% reduction of PSA level after 12 weeks of treatment. There are numerous secondary objectives including time to PSA progression, median metastasis free survival (MFS), and a number of analyses of Hedgehog pathway response, and clinical response.

NCT02357836—A phase 0 study of neo-adjuvant use of ITZ in NSCLC prior to surgical resection. Following base-line assessment of angiogenic and Hedgehog pathway activity patients will receive seven to ten days of oral ITZ at a dose of 600 mg per day, and then be reassessed. This is a pharmacodynamics study looking for evidence of anti-angiogenic and Hedgehog pathway inhibition, in addition to assessing patient’s safety.

NCT02120677—Topical ITZ in the treatment of basal cell carcinoma. This single arm safety study is currently recruiting. Patients are treated with a topical solution of 50% ITZ compounded in petrolatum jelly for between three to seven days. The primary outcome is a measure of Hedgehog (Gli) response. Secondary measures are related to toxicity.

NCT02354261—Basal cell carcinoma nevus syndrome (BCCNS) is a familial cancer pre-disposition syndrome associated with mutations in the Hedgehog signalling pathway. Affected individuals are prone to development of BCC and other cancers. This open-label phase II trial will use a new formulation of ITZ called SUBA-itraconazole at a daily dose of 200 mg (100 mg b.i.d.) in BCCNS patients. The primary outcome is disease response rate. Secondary outcomes are safety and tolerability measures, the duration of responses, and the number of new lesions susceptible to surgical resection.

Additionally, a team at Stanford University is planning to assess the combination of ATO and ITZ in BCC. A current trial, NCT01791894, is investigating the use of ATO. A new trial with the combination is planned for 2015/2016 (Jean Tang, personal communication).

NCT02366884—This single-blinded two-centre phase II trial will utilise a range of anti-bacterial, anti-fungal (including ITZ) and anti-protozoal agents in combinations against all cancer types. The trial is testing a theory that cancer is ‘atavistic’ and shares many of the pathways and mechanisms used by unicellular organisms and that inhibition of these pathways using existing medications for these indications may inhibit cancer. Patient recruitment is aimed at those with terminal disease and no standard of care options. The primary outcome is objective clinical tumour regression rate.

Itraconazole is also a component of the CUSP9* protocol for glioblastoma [37]. This is a multi-agent cocktail of repurposed drugs to be used in addition to standard of care for recurrent glioblastoma. A clinical trial of CUSP9* is currently planned for 2015 (Marc-Eric Halatsch, personal communication).

## Mechanism of action

There are multiple mechanisms of action proposed to explain the diverse anticancer effects of ITZ. These include:

- Anti-angiogenic
- Hedgehog pathway inhibition
- Autophagy induction
- Reversal of multi-drug resistance

### Angiogenesis

A screen by Chong *et al* of FDA approved drugs using a human endothelial cell proliferation assay indicated that ITZ is a potent inhibitor of endothelial cell proliferation at easily achievable plasma levels, with little or no effect on non-endothelial cells [17]. In contrast to other members of the azole anti-fungals (ketoconazole, fluconazole, and voriconazole), ITZ inhibited proliferation, with an IC50 of 0.16 μM. The mechanism appeared to be related to cell cycle arrest at the G1 phase. *In vivo* in a Matrigel mouse model, ITZ administered intraperitoneally with a dose equivalent to human IV dosing, showed a 67.5% decrease of new blood vessel formation compared to untreated controls. The anti-fungal activity of ITZ is because of the inhibition of lanosterol 14α-demethylase (14DM), required to preserve membrane integrity in fungal cells. In humans 14DM is involved in the biosynthesis of cholesterol. *In vitro* the anti-angiogenic effect of ITZ was reduced in the presence of cholesterol. Subsequent analysis has shown that ITZ also caused the inhibition of mammalian target of rapamycin (mTOR) activity and proper cholesterol trafficking in endothelial cells [38].

Rudin and colleagues further investigated the anti-angiogenic activity of ITZ in xenograft models of NSCLC [19]. Immunocompromised mice were injected with LX-14 (squamous cell) and LX-7 (adenocarcinoma) cells derived from primary tumour samples from untreated NSCLC patients. *In vitro* analysis with HUVECs indicated that ITZ inhibited cell migration, chemotaxis, and tube formation. The mouse models were treated with ITZ orally at a dose of 75 mg/kg and both models showed significant reductions in tumour volume. Over a 14 day period ITZ monotherapy in LX-14 and LX-7 mice resulted in 72% and 79% inhibition of tumour growth, respectively, compared to vehicle treated controls (P < 0.001). Combination therapy with cisplatin was superior to cisplatin monotherapy to a statistically significant extent (P ≤ 0.001 compared to ITZ or cisplatin alone) resulting in over 95% growth inhibition but no tumour regression. Treatment with ITZ also decreased tumour vascular area but increased expression of HIF1α.

Further insight into the anti-angiogenic mechanisms of action were provided by investigations into the effects of ITZ on VEGF signalling, specifically showing that ITZ interferes with VEGF binding to VEGFR2 [39]. However, in the metastatic breast cancer trial expression of pro-angiogenic factors (VEGF-A and PlGF) were reduced and levels of the anti-angiogenic factor TSP-1 increased, this was not correlated with drug or metabolite levels [34]. Furthermore the trial in prostate cancer showed no evidence of a change in plasma VEGF levels with ITZ at 200 mg/day and 600 mg/day [27].

However, indirect clinical evidence of anti-angiogenic activity is provided by a case series report in infantile hemangiomas [40]. Physicians treating a case of infantile hemangioma with concurrent fungal infection found that treatment with a range of drugs, including ITZ, resolved the fungal infection and also caused regression of the hemangioma. Subsequent ITZ treatment in five other infants, including four without evidence of fungal infection, also produced regression of lesions.

### Hedgehog inhibition

The Hedgehog pathway is activated in a number of cancer types and is felt to play a major role in the maintenance of cancer stem cells, a relatively chemo- and radio-resistant subset of cancer cells [41]. Thus the notion of treating cancer with agents that target both stem and non-stem cell populations has gained momentum, and ITZ, as a Hedgehog inhibitor, may well play a pivotal role in such therapies. Indeed one indication that ITZ may have such a role comes from *in vivo* work in a multiple myeloma (MM) model in which ITZ increased survival in mice injected with MM stem cells [42].

As noted earlier, initial identification of ITZ as a Hedgehog pathway inhibitor came about via a screen of Food and Drug Administration (FDA) approved drugs [20]. While many drugs were identified as potential Hedgehog pathway inhibitors, few candidates did so at clinically achievable concentrations. In contrast ITZ was shown to have an IC50 of 800 nM, a value approximately ten-fold lower than that of ketoconazole, which had an IC50 of 9 μM. The primary metabolite, hydroxyitraconazole, also inhibited the Hedgehog pathway, with an IC50 of approximately 1.2 μM. Other azole anti-fungals, for example fluconazole, did not show evidence of anti-Hedgehog activity, suggesting that the mechanism of action is not directly related to the anti-fungal activity of this class of drugs. Mechanistic analysis indicated that the anti-Hedgehog activity is via Smoothened, and that it acts in a manner distinct from cyclopamine and other Smoothened antagonists. Later work has investigated the use of ITZ in tumours with acquired resistance to a range of Smoothened inhibitors in clinical development [22].

The finding that ITZ inhibits Hedgehog signalling was confirmed using skin biopsy samples in the phase II randomised trial in men with castration-resistant prostate cancer [27]. A secondary outcome of the trial was an analysis of Hedgehog pathway activity in skin punch biopsies at baseline and at four and 12 weeks after commencement of treatment. GLI1 mRNA expression was used as a marker for Hedgehog pathway activation. Results showed that GLI1mRNA expression was reduced in 33% and 68% of patients in the low- and high-dose arms of the trial. The percentage of patients who showed greater than 50% reduction was 28%, in contrast to 68% in studies using the Hedgehog inhibitor vismodegib. In terms of clinical results, there was a significant correlation between longer PSA PFS and GLI1 reduction (P = 0.028), and a trend towards longer PFS (P = 0.128).

### Autophagy

ITZ was found to induce autophagic cell growth arrest in U87 and C6 glioblastoma cells both *in vitro* and in a mouse xenograft (U87) model [24]. The effect is related to inhibition of mTOR signalling, which is caused by the blockade of cholesterol trafficking by ITZ, as also noted in endothelial cells [38]. ITZ also inhibited AKT1, an upstream regulator of mTOR, and that reactivation of AKT1 reversed the induction of autophagy and growth arrest. Inhibition of autophagy abrogated the reduction of cellular proliferation, suggesting that the use of ITZ with autophagy inhibitors may be problematic.

### Drug resistance

Acquired or multi-drug resistance (MDR) is a common phenomenon in medical treatment, with multiple mechanisms of action including the activity of the drug efflux proteins of the ATP binding cassette (ABC) transporter family. The most well-known of these is P-glycoprotein (P-gp), which is known to be associated with resistance to a wide range of therapeutic agents, including antibiotics and cytotoxic chemotherapy drugs. Reversal of MDR as a clinical strategy to improve response to treatment has been actively investigated for some years [43]. ITZ, in common with some related compounds such as ketoconazole, has been shown to be a potent inhibitor of P-gp at clinically relevant doses [44]. In a cell line over-expressing P-gp, ITZ was able to reduce P-gp transporter function by 50% at a dose of approximately 2 μM.

Another member of the ABC transporter family is human breast cancer resistance protein (BCRP), which is implicated in resistance to a range of active anticancer agents, including anthracyclines, methotrexate, topotecan, mitoxantrone, and other drugs [45]. In an *in vitro* study, HEK cells over-expressing BCRP and resistant to topotecan were treated with a range of azole anti-fungals and tested for efflux of a fluorescent marker [16]. Treatment with ITZ at a dose of 0.1 and 1.0 μM increased intracellular fluorescence by 20% and 40% respectively, showing inhibition of BCRP. ITZ at doses of 2 μM and 5 μM also significantly reversed resistance to the cytotoxic effect of topotecan.

## Our take

The evidence presented above, (and summarised in Table 1), suggests that ITZ has a number of anti-cancer effects at clinically achievable doses, particularly in NSCLC, BCC, and prostate cancer. There is evidence that that it may also be applicable to a number of *other* cancers, including glioblastoma, breast, pancreatic, and ovarian. More generally, the main mechanisms of action investigated to date, antiangiogenic and Hedgehog inhibition, may serve to identify other cancer types where investigation with ITZ may be beneficial.

We note that the experience with many targeted therapies suggest that resistance may become an issue with ITZ as a monotherapy, as it is, for example with another Hedgehog inhibitor, vismodegib [46]. Given the evidence that ITZ acts on a different molecular target to vismodegib, the combination of the two drugs may act to limit the acquisition of such resistance. The combination warrants investigation in a clinical trial with some degree of urgency as vismodegib progresses into clinical use for advanced BCC and other malignancies. There is also intriguing evidence that Hedgehog inhibitors may act to improve immune surveillance and adaptive immune response in BCC, with a suggestion that this may be exploited clinically with the addition of other immunostimulant therapies [47]. Confirmation of this finding in ITZ would add a significant additional mechanism of action with possible clinical benefit and as such further investigation is warranted.

As an ‘old’ drug, it may be that ITZ has multiple molecular targets such that acquired resistance is less of an issue than with agents such as vismodegib which are closely targeted to a single pathway. It would be interesting to combine ITZ with a range of other drugs, including, but not limited to, cytotoxic chemotherapies. The pre-clinical and clinical data with platinum-based chemotherapies and the clinical data with pemetrexed are quite promising. Conceptually, combinations with drugs that target other points in the Hedgehog pathway, or with drugs that target other mechanisms of stem cell survival, or with drugs targeting non-stem cells, all make eminent sense. A number of possible such combinations are outlined in the supplementary materials.

In addition to showing evidence of clinical activity at standard ‘on-label’ therapeutic doses, ITZ can also be used for long periods with generally manageable levels of toxicity. This is particularly important in the context of metronomic chemotherapy schedules, and it may well be that ITZ can serve as a useful adjunct to such protocols. New formulations of ITZ are also being developed commercially which offer greater bioavailability than the standard generic compound. One example is SUBA-Itraconazole (licensed as Lozanoc), developed by Mayne Pharmaceuticals, and being investigated as a possible anti-cancer agent by HedgePath Pharmaceuticals in the trial for BCCNS.

### Next steps

The evidence is strongest for clinical trials of ITZ, in combination with other agents, in the following cancer types:

- NSCLC
- BCC
- Prostate cancer
- Glioblastoma
- Ovarian carcinoma
- Metastatic breast cancer
- Pancreatic cancer

Clinical trials in some of these are on-going, but in others the potential of ITZ is yet to be explored. In particular it should be noted that ITZ could also be of interest in other cancers in which the Hedgehog pathway is known to play an important role. These include rare cancers such as SCLC, medulloblastoma, or certain types of sarcomas (rhabdomyosarcoma, chondrosarcoma, and osteosarcoma), for which very few new drugs are currently being developed, making it an interesting candidate for rapid implementation of clinical trials in these diseases with high unmet needs.

## Conclusion

The evidence for an anti-cancer effect of ITZ treatment comes from *in vitro*, *in vivo*, and human data. It has well-established pharmacokinetics and a known toxicity profile making this generic drug a strong candidate for repurposing as an oncological treatment, both in combination with existing standard of care treatments and with other repurposed drugs. A number of possible multi-drug combinations are outlined, along with their rationale, in the hope that clinicians can initiate clinical trials as a matter of some urgency.

## Author contributions

Primary author: Pan Pantziarka. Contributing authors: Vidula Sukhatme, Gauthier Bouche, Lydie Meheus, Vikas P Sukhatme. All authors have read and approved the final manuscript.

## Competing interests

The authors declare that they have no competing interests. All the authors are associated with not for profit organisations that aim to repurpose drugs for oncology treatments.

## Figures and Tables

**Table 1.** Summary of evidence by cancer type.

Cancer Type	In vitro	In vivo	Case report/trial
Basal cell carcinoma	[20]	[20]	[30] NCT02120677
Prostate			[27], [28] NCT01787331
NSCLC	[19]	[19]	[29] NCT02357836
Ovarian			[31]
Breast cancer triple-negative breast cancer (TNBC)			[33] NCT00798135
Medulloblastoma	[20]		
Glioblastoma	[24]	[24]	[37]
Pancreatic			[35]
