# Supplementary material for: Repurposing Drugs in Oncology (ReDO)—itraconazole as an anti-cancer agent
Source: Ecancermedicalscience. 2015 Apr 15;9:521. doi: 10.3332/ecancer.2015.521 (PMC4406527; doi:10.3332/ecancer.2015.521)
Supplement: Related texts

# Article commentary: Repurposing Drugs in Oncology (ReDO)—itraconazole as an anti-cancer agent–supplementary material

**Published:** 2015

## Introduction

The following drugs warrant further investigation in combination with itraconazole (ITZ) and existing standard of care cancer treatments in a range of cancers. These combinations, listed in Table 1, have been selected on the basis of existing pre-clinical and clinical experience in each of the indications. In some cases these combinations replicate existing protocols currently being tested in clinical trials, but substitute known and repurposed drugs for the newer and/or more toxic agents currently being investigated. All of these proposed combinations are expected to display relatively low toxicity and use low cost and generally available agents. *The following drugs are not listed in order of priority*.

## Cancer stem cells

The following combination therapies are aimed at the elimination of cancer stem cell or tumour-initiating cell populations, in combination with ITZ, via Hedgehog pathway inhibition.

- Fluvastatin—There is both epidemiological and pre-clinical evidence of an anti-cancer effect of lipophilic statins, including simvastatin, lovastatin, and fluvastatin. Multiple mechanisms of action have been proposed and these are summarised in [1]. A number of clinical trials are also currently being carried out investigating the addition of statins to existing standard of care treatments in a wide range of cancers, including prostate, breast, NSCLC, glioblastoma, colorectal, and gastric cancers. There is evidence that concurrent use of ITZ and some statins, such as simvastatin and lovastatin, causes increased toxicity, particularly increased rhabdomyolysis [2, 3]; however, this increased toxicity is not apparent with fluvastatin [2]. Clinical trials of fluvastatin as an anti-cancer agent include for early stage breast cancer, prostate cancer, and optochiasmatic gliomas. Evidence exists that fluvastatin exerts an anti-proliferative effect via induction of autophagy [4, 5]. There is also *in vitro* evidence that fluvastatin acts directly on stem-like cell populations [4]. The combination with ITZ may exert a synergistic pro-autophagic effect, particularly in glioblastoma and other malignancies where autophagy has an anti-tumour effect.
- Mebendazole—There is a range of pre-clinical and clinical evidence of an anti-cancer effect for the widely used anti-helminthic drug mebendazole. There are a number of distinct possible mechanisms of action and the drug currently being assessed in a number of clinical trials as an anti-cancer agent [6]. Mebendazole is known to act as a microtubule-disrupting agent to achieve its anti-parasitic action, and it is suggested that in part this may also play a role in the anti-cancer activity. However, there is also evidence that mebendazole is anti-angiogenic [7] and possibly act on the Hedgehog pathway [8]. The combination with ITZ, which has complementary anti-angiogenic and anti-Hedgehog properties is appealing and may be of clinical benefit in NSCLC, glioblastoma, medulloblastoma, prostate, and ovarian cancer.
- Disulfiram—Long used as a treatment for alcohol abuse, disulfiram is now undergoing extensive investigation as a possible agent to target cancer stem cell populations [9–11]. Other possible mechanisms of action include direct pro-apoptotic effects [12], anti-MMP activity [13], and reversal of drug resistance [14]. In addition to extensive pre-clinical data, disulfiram is undergoing investigation in a number of clinical trials including in glioblastoma, pancreatic, colorectal, and breast cancer. The combination with ITZ may be especially useful in glioblastoma, for which there is extensive pre-clinical evidence of activity. It should be noted that ITZ and disulfiram are both included in the innovative CUSP9* protocol for recurrent GBM [15].

## Cancer stem and non-stem cells

The following combinations with ITZ target both stem and non-stem cell populations within tumours. Since existing evidence indicates that ITZ is synergistic with existing cytotoxic chemotherapy drugs including platinum-based agents and the anti-folate pemetrexed, additional investigation of these combinations is clearly warranted. There are some indications, based on *in vitro* and *in vivo* analysis, that Hedgehog signalling is implicated in treatment failure in radiotherapy, and that Hedgehog inhibition may be beneficial in combination with radiotherapy and chemo-radiation protocols [16, 17]. Therefore investigation of ITZ as a potential adjunct to radiotherapy is justified.

- Methotrexate—Early stage positive results have been reported for the combination of ITZ and the anti-folate chemotherapeutic pemetrexed in NSCLC [18]. Methotrexate, a commonly used first generation anti-folate drug, has yet to be explored in combination with ITZ. Methotrexate is used to treat a variety of cancers in both maximum tolerate dose (MTD) and metronomic dosing schedules and is one of the core drugs for the treatment of bone and soft tissue sarcomas. There is also increasing evidence that aberrant Hedgehog signalling exists in sarcomas [19], suggesting that ITZ may have some potential in treatment. Therefore, given the similar mechanism of action to pemetrexed and the existing efficacy of methotrexate in sarcomas, the combination with ITZ deserves further clinical investigation.

## Anti-angiogenic/immunological

The following combinations with ITZ seek to exploit anti-angiogenic and/or immune-related activity rather than cytotoxicity.

- Metronomic chemotherapy—In contrast to MTD chemotherapy, there is evidence that low-dose metronomic chemotherapy acts primarily through non-cytotoxic means. A variety of existing chemotherapeutic agents have been successfully used in metronomic protocols, including oral cyclophosphamide, capecitabine, methotrexate, etoposide, vinorelbine, and temozolomide [20]. There is evidence that the combination of metronomic chemotherapy with additional anti-angiogenic agents may be beneficial [21]. ITZ inhibits at least two angiogenic pathways, as evidenced by its ability to block VEGF and FGF-induced angiogenesis [22]. There is also a suggestion that inhibition of drug efflux may reduce the risk of acquired resistance to some metronomic treatments [23]. Therefore, as there is evidence that ITZ is both anti-angiogenic and inhibits P-gp, it warrants clinical study in combination with metronomic chemotherapy schedules.
- Celecoxib—The NSAID celecoxib is undergoing clinical investigation in a number of clinical trials in a range of cancer types, including colorectal, basal cell carcinoma, prostate, NSCLC, and paediatric brain tumours. Positive results of the combination of celecoxib and standard of care treatments have been reported in a range of cancers, including prostate [24], colorectal [25], ovarian [26] and for chemo-prevention in high-risk non-melanoma skin cancer [27]. While there are some concerns regarding the effects of ITZ on the pharmacokinetics of NSAIDs, a study in healthy human volunteers showed no significant changes in celecoxib bioavailability with concurrent ITZ [28]. Based on the evidence for both ITZ and celecoxib, the combination warrants clinical investigation in a wide range of cancer types, including basal cell carcinoma, prostate cancer, glioblastoma, colorectal, and ovarian cancers.
- Cimetidine—The well-known H_2_RA, cimetidine is primarily used to treat peptic ulcers and heartburn, but has also been shown to have pre-clinical and clinical evidence of anti-cancer activity in different cancers [29, 30]. A number of mechanisms of action have been proposed, including effects on cell adhesion, angiogenesis, and a range of immunomodulatory effects. The strongest clinical evidence shows a positive effect on survival when used peri-operatively for early stage colorectal cancer [31]. These anti-cancer effects may enhance the activity of ITZ, particularly with respect to cimetidine’s immunomodulatory effect. Given the proposed evidence of a positive effect on immune surveillance induced by oral Hedgehog inhibitors, the combination with cimetidine should be investigated in a suitable animal model [32]. Caution must be exercised however, as the suppression of gastric acid induced by cimetidine may interfere with the pharmacokinetics of ITZ. One strategy to ameliorate the issue is to administer the ITZ tablets with an acidic drink [33]. However, additional pre-clinical work in an appropriate animal model may be warranted before moving to a clinical trial.

## Figures and Tables

**Table 1.** Proposed drug combinations with ITZ and standard of care in different cancers.

Disease	Targets	Drug combination
Glioblastoma	Decrease proliferation and increase apoptosis via induction of autophagy.	Fluvastatin [4]
		Celecoxib
		Mebendazole
		Disulfiram (NCT01907165)
Basal cell carcinoma	Hedgehog and COX-2 inhibition	Celecoxib
Prostate	Hedgehog inhibition, anti-angiogenic, microtubule disruption	Fluvastatin
		Mebendazole [34]
		Metformin (*NCT01561482*)
NSCLC	Increase chemo/radio-sensitivity, improve enhance permeability and retention (EPR) effect, AMP-activated protein kinase (AMPK)/mTOR, COX-2 inhibition, and immunomodulation	Metformin (NCT01997775)
		Celecoxib (NCT00520845)
		Mebendazole
Melanoma	Microtubule disruption, anti-angiogenic, anti-inflammatory, COX-2	Diclofenac or celecoxib [35]
		Mebendazole [36]
Colorectal	Microtubule disruption, AMPK/mTOR, immunomodulation, anti-histamine,COX-2	Mebendazole [37]
		Metformin (NCT01941953)
		Aspirin [38]
		Cimetidine [31]
Ovarian	Anti-angiogenic, anti-inflammatory, microtubule disruption	Metronomic cyclophosphamide [39]
		Mebendazole
		Cimetidine [40]

Note that references to clinical trials or published papers are indicative of trials or case reports where the drug (or analogue) has been used for the specific indication.

## References

[1] Osmak M (2012). Statins and cancer: current and future prospects. Cancer Lett.

[2] Kivistö KT, Kantola T, Neuvonen PJ (2012). Different effects of itraconazole on the pharmacokinetics of fluvastatin and lovastatin. Br J Clin Pharmacol.

[3] Neuvonen PJ, Kantola T, Kivistö KT (1998). Simvastatin but not pravastatin is very susceptible to interaction with the CYP3A4 inhibitor itraconazole. Clin Pharmacol Ther.

[4] Jiang P (2014). In vitro and in vivo anticancer effects of mevalonate pathway modulation on human cancer cells. Br J Cancer.

[5] Qi X-F (2013). Autophagy contributes to apoptosis in A20 and EL4 lymphoma cells treated with fluvastatin. Cancer Cell Int.

[6] Pantziarka P (2014). Repurposing Drugs in Oncology (ReDO)-mebendazole as an anti-cancer agent. Ecancermedicalscience.

[7] Bai R-Y (2015). Effective treatment of diverse medulloblastoma models with mebendazole and its impact on tumor angiogenesis. Neuro Oncol.

[8] Larsen AR (2014). Repurposing the antihelmintic mebendazole as a hedgehog inhibitor. Mol Cancer Ther.

[9] Triscott J, Pambid M, Dunn S (2012). Bullseye: Targeting cancer stem cells to improve the treatment of gliomas by repurposing Disulfiram. Stem Cells (Dayton, Ohio).

[10] Hothi P (2012). High-throughput chemical screens identify disulfiram as an inhibitor of human glioblastoma stem cells. Oncotarget.

[11] Wang Y (2014). Blocking the formation of radiation-induced breast cancer stem cells. Oncotarget.

[12] Cheriyan VT (2014). Disulfiram suppresses growth of the malignant pleural mesothelioma cells in part by inducing apoptosis. PLoS ONE.

[13] Cho HJ (2007). Disulfiram suppresses invasive ability of osteosarcoma cells via the inhibition of MMP-2 and MMP-9 expression. J Biochem Mol Biol.

[14] Liu P (2013). Disulfiram targets cancer stem-like cells and reverses resistance and cross-resistance in acquired paclitaxel-resistant triple-negative breast cancer cells. Br J Cancer.

[15] Kast RE, Karpel-Massler G, Halatsch M (2012). CUSP9* treatment protocol for recurrent glioblastoma: aprepitant, artesunate, auranofin, captopril, celecoxib, disulfiram, itraconazole, ritonavir, sertraline augmenting continuous low dose temozolomide. Oncotarget.

[16] Ma J (2013). Sonic Hedgehog Signaling Pathway Supports Cancer Cell Growth during Cancer Radiotherapy. PLoS ONE.

[17] Chaudary N (2012). Hedgehog pathway signaling in cervical carcinoma and outcome after chemoradiation. Cancer.

[18] Rudin CM (2013). Phase 2 study of pemetrexed and itraconazole as second-line therapy for metastatic nonsquamous non-small-cell lung cancer. J Thorac Oncol.

[19] Kelleher FC (2012). Prevailing importance of the hedgehog signaling pathway and the potential for treatment advancement in sarcoma. Pharmacol Ther.

[20] André N, Carré M, Pasquier E (2012). Metronomics: towards personalized chemotherapy?. Nat Rev Clin Oncol.

[21] Digklia A, Voutsadakis IA (2012). Combinations of vascular endothelial growth factor pathway inhibitors with metronomic chemotherapy: Rational and current status. World J Exp Med.

[22] Aftab BT (2011). Itraconazole inhibits angiogenesis and tumor growth in non-small cell lung cancer. Cancer Res.

[23] Akiyama K (2014). Inhibition of Multidrug Transporter in Tumor Endothelial Cells Enhances Antiangiogenic Effects of Low-Dose Metronomic Paclitaxel. American J Pathol.

[24] Sooriakumaran P (2009). A randomized controlled trial investigating the effects of celecoxib in patients with localized prostate cancer. Anticancer Res.

[25] Jin C-H (2011). Observation of curative efficacy and prognosis following combination chemotherapy with celecoxib in the treatment of advanced colorectal cancer. J Int Med Res.

[26] Legge F (2011). Phase II study of the combination carboplatin plus celecoxib in heavily pre-treated recurrent ovarian cancer patients. BMC Cancer.

[27] Elmets CA (2010). Chemoprevention of nonmelanoma skin cancer with celecoxib: a randomized, double-blind, placebo-controlled trial. J Natl Cancer Inst.

[28] Jayasagar G (2003). Effect of itraconazole on the pharmacokinetics of celecoxib in healthy human volunteers. Pharmazie.

[29] Pantziarka P (2014). Repurposing drugs in oncology (ReDO)-cimetidine as an anti-cancer agent. Ecancermedicalscience.

[30] Kubecova M (2011). Cimetidine: an anticancer drug?. Euro J Pharm.

[31] Deva S, Jameson M (2012). Histamine type 2 receptor antagonists as adjuvant treatment for resected colorectal cancer. The Cochrane Database Syst Rev.

[32] Otsuka A (2015). Hedgehog pathway inhibitors promote adaptive immune responses in basal cell carcinoma. Clin Cancer Res.

[33] Oguma T (2009). Influence of black vinegar on itraconazole absorption. Kansenshōgaku Zasshi.

[34] Briasoulis E (2013). Dose selection trial of metronomic oral vinorelbine monotherapy in patients with metastatic cancer: a hellenic cooperative oncology group clinical translational study. BMC Cancer.

[35] Bhatt RS (2010). A phase 2 pilot trial of low-dose, continuous infusion, or “metronomic” paclitaxel and oral celecoxib in patients with metastatic melanoma. Cancer.

[36] Doudican NA (2013). XIAP downregulation accompanies mebendazole growth inhibition in melanoma xenografts. Anticancer Drugs.

[37] Nygren P, Larsson R (2012). Drug repositioning from bench to bedside: Tumour remission by the antihelmintic drug mebendazole in refractory metastatic colon cancer. Acta Oncol.

[38] Bastiaannet E (2012). Use of Aspirin postdiagnosis improves survival for colon cancer patients. Br J Cancer.

[39] Barber EL (2013). The combination of intravenous bevacizumab and metronomic oral cyclophosphamide is an effective regimen for platinum-resistant recurrent ovarian cancer. J Gynecol Oncol.

[40] Niwa K (2008). Prognostic implications of cimetidine on advanced serous ovarian carcinoma related to cyclooxygenase-2 expression. Molecular Med Rep.
